# Construction and validation model of necroptosis-related gene signature associates with immunity for osteosarcoma patients

**DOI:** 10.1038/s41598-022-20217-4

**Published:** 2022-09-23

**Authors:** Long Hua, Pengfei Lei, Yihe Hu

**Affiliations:** 1 Department of Orthopedics, Xiangya Hospital Central South University, Changsha, Hunan People’s Republic of China; 2 Department of Orthopedics, The First Affiliated Hospital, Medical College of Zhejiang University, Hangzhou, People’s Republic of China; 3 Department of Orthopedics, The Sixth Affiliated Hospital, Xinjiang Medical University, Ürümqi, People’s Republic of China

**Keywords:** Cancer, Bone cancer, Cancer models, Cancer prevention, Cancer therapy

## Abstract

Osteosarcoma is the most common malignant tumor in children and adolescents and its diagnosis and treatment still need to be improved. Necroptosis has been associated with many malignancies, but its significance in diagnosing and treating osteosarcoma remains unclear. The objective is to establish a predictive model of necroptosis-related genes (NRGs) in osteosarcoma for evaluating the tumor microenvironment and new targets for immunotherapy. In this study, we download the osteosarcoma data from the TARGET and GEO websites and the average muscle tissue data from GTEx. NRGs were screened by Cox regression analysis. We constructed a prediction model through nonnegative matrix factorization (NMF) clustering and the least absolute shrinkage and selection operator (LASSO) algorithm and verified it with a validation cohort. Kaplan–Meier survival time, ROC curve, tumor invasion microenvironment and CIBERSORT were assessed. In addition, we establish nomograms for clinical indicators and verify them by calibration evaluation. The underlying mechanism was explored through the functional enrichment analysis. Eight NRGs were screened for predictive model modeling. NRGs prediction model through NMF clustering and LASSO algorithm was established. The survival, ROC and tumor microenvironment scores showed significant statistical differences among subgroups (P < 0.05). The validation model further verifies it. By nomogram and calibration, we found that metastasis and risk score were independent risk factors for the poor prognosis of osteosarcoma. GO and KEGG analyses demonstrate that the genes of osteosarcoma cluster in inflammatory, apoptotic and necroptosis signaling pathways. The significant role of the correlation between necroptosis and immunity in promoting osteosarcoma may provide a novel insight into detecting molecular mechanisms and targeted therapy.

## Introduction

Osteosarcoma is the most common bone malignant tumor in children, with a high lung metastasis rate, easy recurrence and poor prognosis^1,2^. The current adolescent incidence rate is 0.0004–0.0005%^3^. Traditional treatments such as surgical resection, radiotherapy and chemotherapy are still challenging to reduce the mortality of osteosarcoma^4–6^. This is because osteosarcoma is challenging to diagnose early and has a highly heterogeneous and complex cancer, making it difficult to cure^7,8^. Therefore, how to make an early diagnosis and treatment of osteosarcoma is still an urgent problem and challenge for researchers.

Necroptosis is a type of programmed necronecrosis. In recent years, it has been one of the tens of light-years identified to regulate receptor-kinases (RIPK1/RIPK3) and mixed lineage kinase domain-like proteins (MLKL) specific cell death patterns^9–11^. It has been proved to cause cell death and crosstalk with inflammatory response, a momentous event regulating tumor occurrence and progression^12,13^. Therefore, necroptosis may be a promising new target for treating osteosarcoma. Some scholars have found that Emodin can mediate glioma cell apoptosis through TNF-α/RIP1/RIP3 pathway^14^. Moreover, necroptosis strategies have been reported to treat osteosarcoma disease^15–18^. In addition, necroptosis modifies the tumor immune microenvironment by regulating immune checkpoints^19^. Last, necroptosis-related lncRNA and miRNAs gene sets have been reported to predict lung cancer prognosis^20,21^. However, whether necroptosis-related genes (NRGs) play a role in the occurrence and development of osteosarcoma disease is still unknown. As such, the prediction of NRG signature regulating osteosarcoma to influence further the mechanism of tumor immunity microenvironment (TIME) has excellent application prospects.

In this study, we download the database of OS patients from TARGET and GEO and the expression profile of normal muscle tissue from GTEx. NRGs were screened by Cox regression analysis. Through nonnegative matrix factorization (NMF) clustering and the least absolute shrinkage and selection operator (LASSO) algorithm to calculate a risk score, a prediction model with NRGs signature for the training cohort is established and verified with the validation cohort. Kaplan–Meier (KM) survival time, Receiver Operating Characteristic (ROC), tumor invasion microenvironment and CIBERSORT were assessed. Establish nomograms for clinical indicators and verify them by calibration evaluation. Finally, through the functional enrichment to explore the underlying mechanism. The objective is to establish a predictive model of NRGs in osteosarcoma for evaluating tumor microenvironment and new targets for immunotherapy.

## Materials and methods

### Data acquisition of osteosarcoma

**Figure 1:**
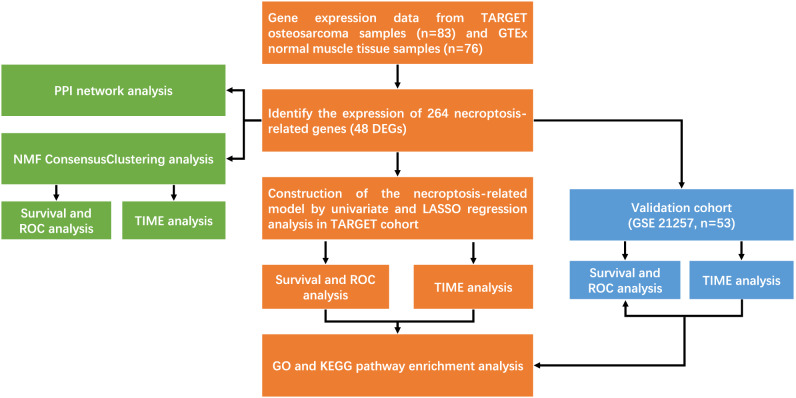
Flow chart of the study.

We obtained the dataset from the TARGET (Therapeutically Applicable Research to Generate Effective Treatments; https://ocg.cancer.gov/programs/target) and GEO (Gene Expression Omnibus) database of NCBI (www.ncbi.nlm.hih.gov/gds). Dataset of osteosarcoma from TARGET contained 83 samples and dataset GSE21257 included 53 samples. NRGs were obtained from Genecards and finally, 48 NRGs were obtained. In addition, the GTEx (Genotype-Tissue Expression; https://xenabrowser.net/) dataset of normal muscle tissue (n = 76) was used for the control group for functional clustering analysis (Table 1). The flow analysis chart is as follows (Fig. 1).

**Table 1:** Characteristics of patients in training and verification cohorts.

Characteristic	Training cohort (n = 83)	Validation cohort (n = 53)
Age, mean ± SD	14.54 ± 3.81	18.71 ± 12.20
**Gender, no. (%)**		
Female	37 (45%)	19 (36%)
Male	46 (55%)	34 (64%)
**Metastasis, no. (%)**		
No	61 (73%)	19 (36%)
Yes	22 (27%)	34 (64%)
**Event, no. (%)**		
None	33 (40%)	NA
Relapse	37 (45%)	NA
Dead	2 (2%)	NA
Other	11 (13%)	NA
**Tumor. location, no. (%)**		
Arm	7(8%)	7 (13%)
Leg	72(87%)	44 (83%)
Other	4(5%)	2 (4%)
**Huvos. grade, no. (%)**		
1	NA	13 (25%)
2	NA	16 (30%)
3	NA	13 (25%)
4	NA	5 (9%)
Unknown	NA	6 (11%)

### NRGs screening and NMF cluster analysis

We used Cox regression analysis to screen NRGs to establish the predictive model. Protein–protein interaction predictions for NRGs were made using the STRING database (https://cn.string-db.org/)^22^. The results were visualized by Cytoscape software^23^ and hub genes were extracted by Cytohubba^24^. The osteosarcoma patients of TARGET were clustered by NMF package of the R language and KM survival analysis was performed according to the results. Estimated Stromal and Immune cells in Malignant Tumor tissues using Expression data were used to analyze the stromal score, immune score, ESTIMATE score and tumor purity of two clusters. The immune cell infiltration score and percentage were analyzed using CIBERSORT algorithms to assess immune infiltration^25,26^.

### Establishment and verification of NRGs prediction model

First, NRGs of osteosarcoma patients in the TARGET database were further screened. We use the glmnet package in R language to build a prediction model of genes by the LASSO algorithm. Necroptosis-related genes were statistically significant in univariate and multivariate analyses. Therefore, the risk score formula is obtained, which is risk score = *β*_1_*X*_1_ + *β*_2_*X*_2_ + *⋯* + *β*_*n*_*X*_*n*_, where *X*_1_*, X*_2_*, ⋯, X*_*n*_ is the corresponding predictor and *β* is the corresponding regression coefficient. Eight necroptosis-related genes were selected to create a predictive model. These genes were MYCT1, BNIP3L, LRP1, OPTN, TRIP6, ATF4, TNFRSF1A and CLTCL1 (Table 2). The risk score formula was constructed as risk score = (0.21 × MYCT1 expression) + (0.19 × BNIP3L expression) + (0.13 × LRP1 expression) + (0.11 × OPTN expression) + (0.02 × TRIP6 expression) + (0.005 × ATF4 expression) + (−0.07 × TNFRSF1A expression) + (−0.94 × CLTCL1 expression). Cox regression analysis algorithm was used to score the risk of each patient. Samples were divided into high-risk and low-risk groups, with the median as the dividing line. We use this algorithm to validate the GEO data set as the validation set.

**Table 2:** Genes included for construction prognostic-related gene signature.

Sig. genes	Coef	Full names	Category	Genecard ID
MYCT1	0.210456925	MYC target 1	Protein coding	GC06P152697
BNIP3L	0.194295857	BCL2 interacting protein 3 like	Protein coding	GC08P026296
LRP1	0.131815591	LDL receptor related protein 1	Protein coding	GC12P057128
OPTN	0.112333038	Optineurin	Protein coding	GC10P013099
TRIP6	0.020655937	Thyroid hormone receptor interactor 6	Protein coding	GC07P100867
ATF4	0.005482178	Activating transcription factor 4	Protein coding	GC22P039599
TNFRSF1A	−0.065762566	TNF receptor superfamily member 1A	Protein coding	GC12M006328
CLTCL1	−0.944261267	Clathrin heavy chain like 1	Protein coding	GC22M019266

### Independence detection of the risk prediction model

Univariate and multivariate Cox regression analyses of independent prognostic-related factors were conducted. Survival curves and risk scores were carried out between different ages, gender and metastasis status. The prerequisite for using the Cox regression model is that the strength of the effect of risk factors on the risk of death is consistent over time.

### Construction and calibration of the nomogram

A nomogram through the LASSO prediction model includes clinical characteristics such as age, gender, metastasis and risk level. Calibration at 1, 3, 5 year survival was carried out in the training cohort and verified in the validation cohort. Further, we evaluated the consistency between the predicted model and the actual observed survival values by mapping a calibration line.

### Differentially expressed genes (DEGs) and functional analyses

The potential efficacy of NRGs in osteosarcoma patients was explored through functional enrichment analysis. NRGs were extracted from the osteosarcoma and normal tissue. Gene Ontology (GO) and Kyoto Encyclopedia of Genes and Genomes (KEGG) analysis were performed on DEGs using Clusterprofiler R Package^27–29^. NRG clustering results, protein–protein interaction (PPI) and hub genes were verified by Metascape (http://metascape.org/gp/).

### Statistical analyses

R language Software (version 3.3.4, The R Foundation for Statistical Computing) and Prism 8 (GraphPad Software, USA) were used for plotting and analysis. Survival analysis was statistically analyzed using the KM survival curve and log-rank analysis. The data were tested for normal distribution. The Student’s *t* test was used for statistical comparison between the two groups conforming to the normal distribution. One-way analysis of variance (ANOVA) is used for statistical comparison between groups that conform to normal distribution. Wilcox nonparametric test was used for data that did not conform to normal distribution. P < 0.05 was considered statistically significant.

## Results

### Construction of prognostic-related NRGs

**Figure 2:**
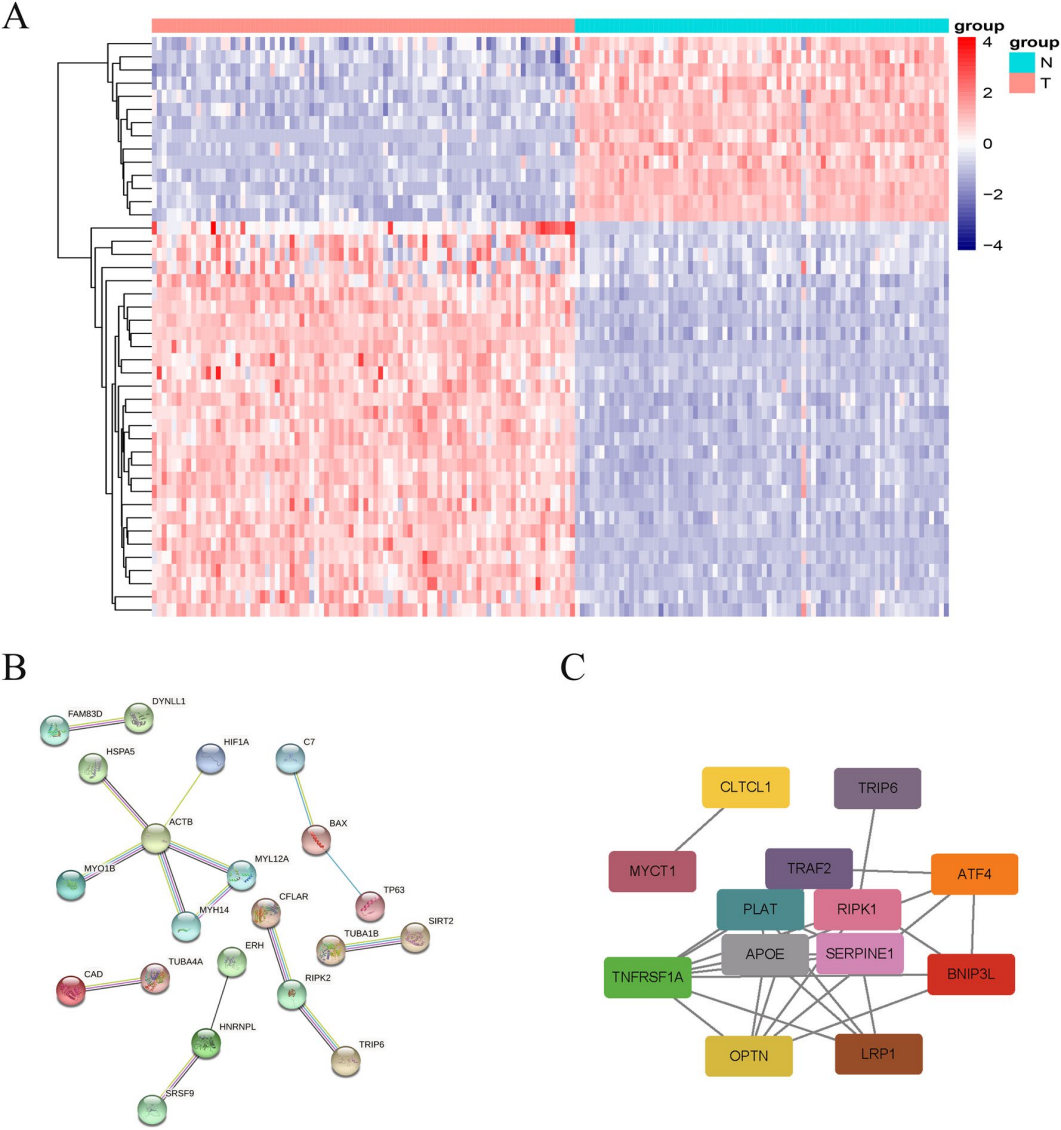
Necroptosis-related different genes and protein–protein interaction predicting. (**A**) Heatmap of differential genes. (**B**) Indicating protein–protein interaction (**C**) Hub genes screening based on protein interaction.

Forty-eight necroptosis-related differential genes were screened and compared with the TARGET database OS and the GTEx normal muscle tissue groups. The results are visualized through heat maps (Fig. 2A). PPI prediction analysis was conducted on NRGs through the STRING website and the results were visualized (Fig. 2B). Eight genes were screened out by the LASSO algorithm and Cox regression analysis as the prediction model signature (Table 2). The interaction between hub genes and protein was predicted and visualized by Cytoscape software (Fig. 2C).

### Establishment and verification of NRGs prediction model

**Figure 3:**
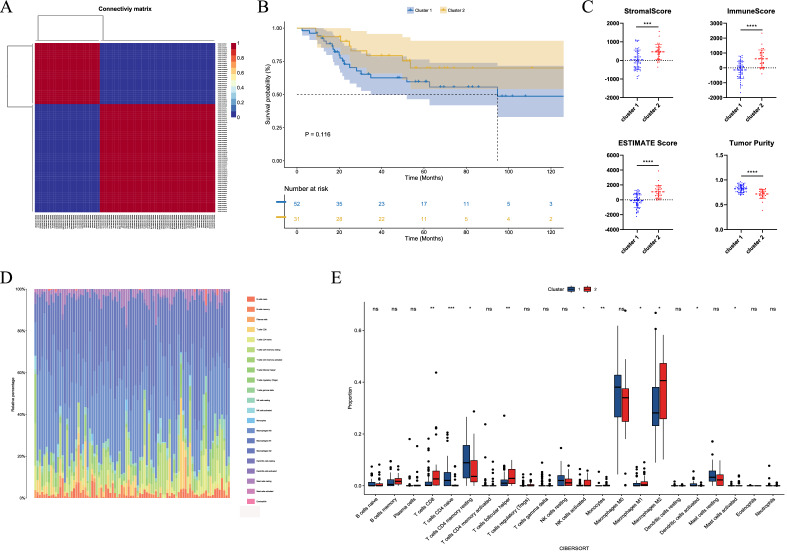
Analyzing classification of subgroups based on nonnegative matrix factorization (NMF) cluster. (**A**) NMF divides the expressed values into two subgroups of matrices. (**B**) Survival curve of the patients based on two clusters. (**C**) Stromal score, immune score, ESTIMATE score and tumor purity compared by the two clusters. (**D**) Assess the proportion of inflammatory cells by CIBERSORT. (**E**) Inflammatory cell infiltration analysis by CIBERSORT.

To verify whether there are differences in the classification of OS patients based on NRGs, analyze a variety of subgroups based on NMF clusters. NMF results showed that two clusters were ideal grouping methods (Fig. S1). Therefore, the expressed values of the patient are selected into two subgroups by an NMF matrix (Fig. 3A). The survival curve of the patients based on two clusters showed a significant difference in survival time between the two groups (Fig. 3B). We performed ESTIMATE analysis of the intergroup tumor cell microenvironment. It was found that there were significant differences in the stromal score, immune score, ESTIMATE score and tumor purity compared by the two clusters between the two groups (Fig. 3C). We then assessed the proportion and differences of specific 22 types of immune cells by CIBERSORT. T cells CD8, T cells CD4 naive, T cells CD4 memory resting, T cells follicular helper, NK cells activated, monocytes, macrophages M1, macrophages M2, dendritic cells activated, mast cells activated groups were significantly different (Fig. 3D,E).

### Construction of predictive model based on the TARGET dataset

**Figure 4:**
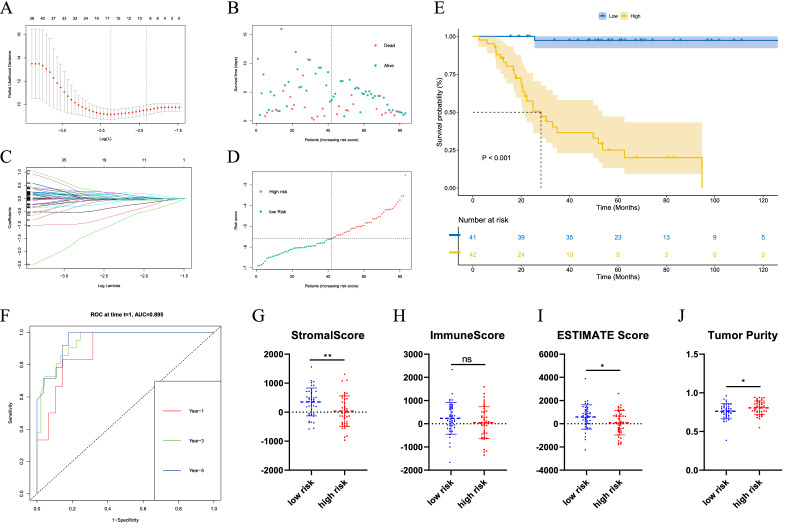
Construction of the prediction model through the LASSO algorithm. (**A**) Selection of the optimal penalty parameter for LASSO regression. (**B**) Survival status and time distribution in the prediction cohort. (**C**) LASSO Cox regression analysis. (**D**) The prediction risk score distribution. (**E**) Survival curve of the high and low-risk groups. (**F**) Time-dependent ROC curve of 1, 3, 5 years. (**G**–**J**) The two groups compared stromal score, immune score, ESTIMATE score and tumor purity.

To verify the prediction of NRG results, we constructed a prediction model through the LASSO algorithm. The optimal penalty parameter was selected by LASSO regression analysis (Fig. 4A). Samples were displayed through Survival status and time distribution in the Prediction cohort (Fig. 4B). LASSO Cox Regression analysis shows the minimum Lamda values (Fig. 4C). The prediction risk score distribution is further analyzed (Fig. 4D). After establishing the prediction model based on the above analysis, we further investigated the survival curve of the high and low-risk groups and the results showed that the survival time of the high-risk group was significantly lower than that of the low-risk group, with a statistical difference (P < 0.05) (Fig. 4E). We then evaluated the prediction efficiency of 1, 3 and 5 years by time-dependent ROC curve and calculated the areas under curve (AUC) results of 89.46, 94.42 and 95.47%, respectively (Fig. 4F). Meanwhile, the two groups compared stromal score, immune score, ESTIMATE score and tumor purity (Fig. 4G–J). The results showed that the value of the stromal score and ESTIMATE score in the low-risk group was higher than that in the high-risk group, while the tumor purity in the low-risk group was lower than that in the high-risk group (P < 0.05). The immune score showed no significant statistical difference between the two groups. CIBERSORT immune cell infiltration was further performed and no significant difference was observed between the groups (Fig. S2).

### Independence detection of the risk prediction model

**Figure 5:**
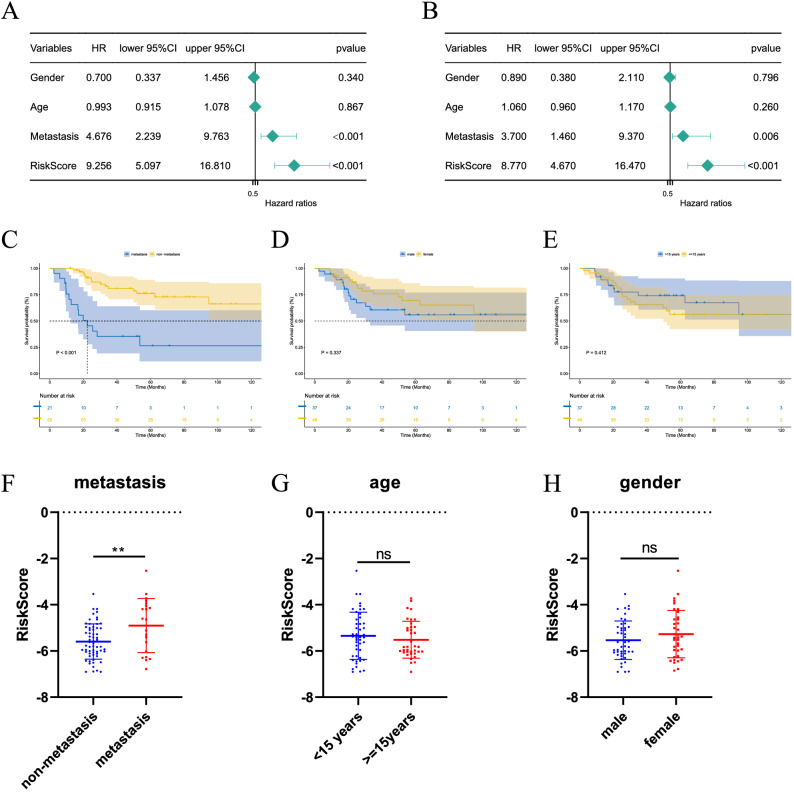
Independence detection of the risk prediction model. (**A**,**B**) Univariate and multivariate Cox regression analysis of independent prognostic-related factors. (**C**–**E**) Survival curves with different age, gender and metastasis status. (**F**–**H**) Risk scores with different ages, gender and metastasis status.

We performed Cox univariate and multivariate regression analyses for clinical indicators such as sex, age, metastasis and risk score, in an attempt to predict outcome variables by clinical indicators. Regardless of univariate or multivariate analysis, metastasis and risk score were independent risk factors for poor prognosis of osteosarcoma regardless of univariate or multivariate analysis. At the same time, age and gender showed no significant statistical difference in the hazard ratio (Fig. 5A,B). Then, we calculated the KM survival curves by subgroups of metastasis, gender and cut-off age at 15. It was found that the metastasis group had a lower survival time than the non-metastasis group (P < 0.001). However, gender and age groups showed no significant difference in survival outcomes (Fig. 5C–E). After that, we evaluated the risk score of different subgroups and found that the risk score of the metastasis group was higher than that of the non-metastasis group (P < 0.01). However, gender and age groups had no significant difference in risk score results (Fig. 5F–H).

### Verification of the predictive model through the GEO cohort

**Figure 6:**
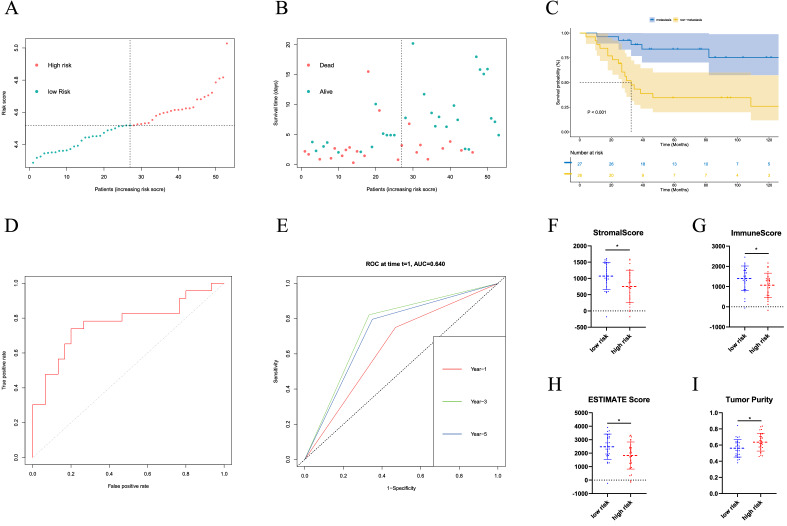
Validation of the prognostic factors in the verification cohort. (**A**,**B**) Distribution of survival status and risk score in the validation cohort (**C**) Survival curve in the validation cohort. (**D**) ROC curve of the prediction model in the validation cohort (**E**) Time-dependent ROC curve of 1, 3, 5 year in the validation cohort. (**F**–**I**) The two groups compared stromal score, immune score, ESTIMATE score and tumor purity.

We verify the efficacy of the prediction model with GSE21257 of the GEO dataset. Patients were grouped into two groups using a predictive model. Samples were displayed through survival status and time distribution in the prediction cohort (Fig. 6A). The prediction risk score distribution is further analyzed with a relatively scattered distribution (Fig. 6B). We further verified the survival curve of the high and low-risk groups and the results showed that the survival time of the high-risk group was significantly lower than that of the low-risk group, with statistical differences (P < 0.001) (Fig. 6C). Furthermore, we verified the prediction efficiency of the model through the ROC curve and calculated the AUC of the subline area was 77.1% (Fig. 6D). Then we evaluated the prediction efficiency of 1, 3 and 5 years by time-dependent ROC curve and calculated the areas under curve (AUC) results of 64.12, 76.65 and 74.53%, respectively (Fig. 6E). The two groups compared stromal score, immune score, ESTIMATE score and tumor purity (Fig. 6F–I). The results showed that the value of the stromal score and ESTIMATE score in the low-risk group was higher than that in the high-risk group, while the tumor purity in the low-risk group was lower than that in the high-risk group (P < 0.05).

### Nomogram established and validated in datasets

**Figure 7:**
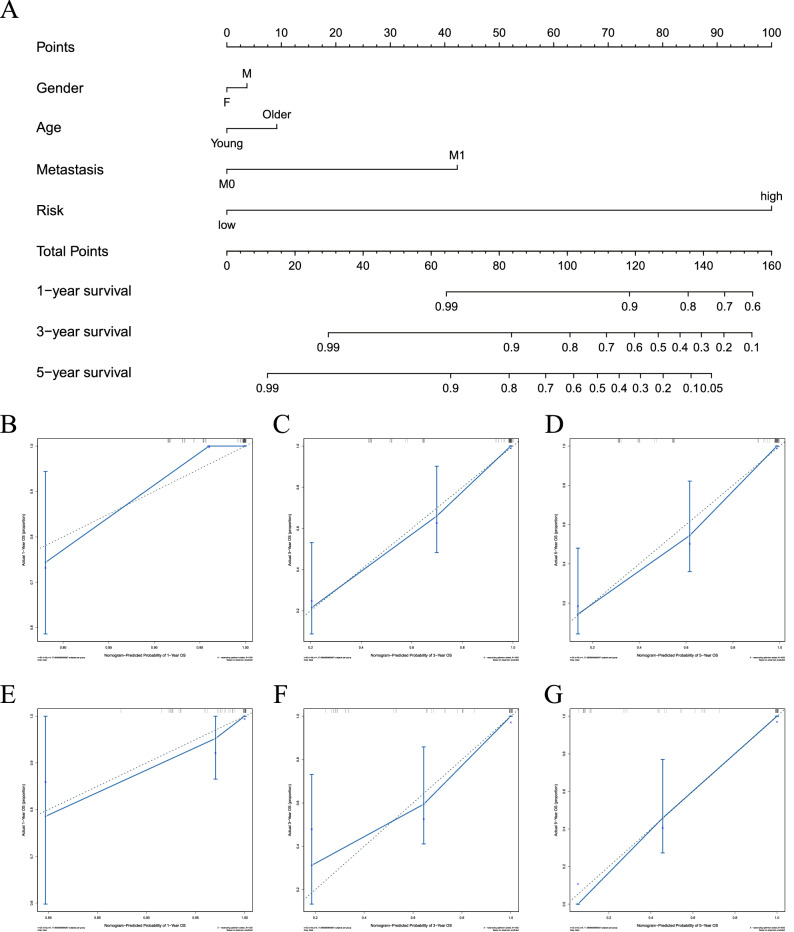
Construction and calibration of the nomogram. (**A**) Nomogram includes clinical characteristics and risk level. (**B**–**D**) Calibration at 1, 3, 5 year survival in the training cohort. (**E**–**G**) Calibration at 1, 3, 5 year survival in the validation cohort.

We constructed a nomogram by searching for critical clinical indicators such as gender, age, metastasis and risk prediction group through the data set of osteosarcoma patients and predicting the outcome variables such as survival time and final status (Fig. 7A). It can be seen that the total points scored by the above clinical indicators correspond to the 1, 3 and 5 year survival rates. Then we predicted the results of the training cohort and validation cohort. In the calibration picture, the blue line represents the data result of actual observation and the gray line represents the survival result predicted by our model. It can be seen that the two lines have good consistency at 1, 3 and 5 years, indicating that the prediction model is ideal (Fig. 7B–D). Similarly, we tested GEO data and found that the observed data fit well with the predicted data (Fig. 7E–G).

### Functional annotations of NRGs

**Figure 8:**
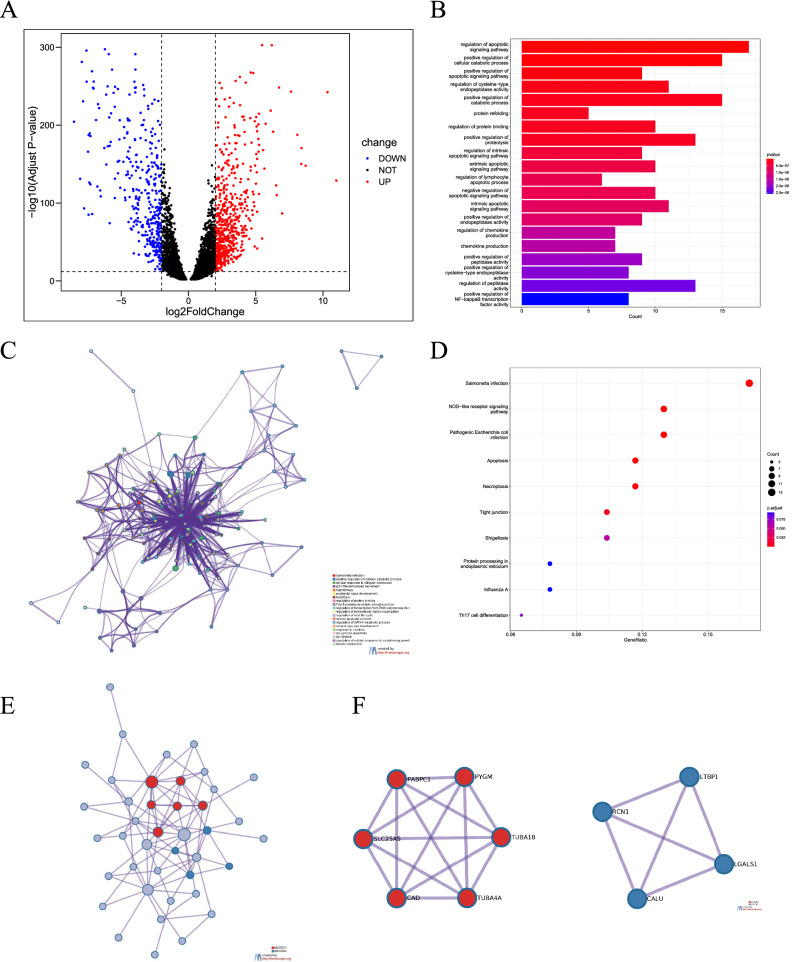
Differentially expressed genes and enrichment analyses. (**A**) Volcano plot showing the necroptosis-related DEGs between the osteosarcoma and normal tissue. (**B**,**C**) Gene Ontology (GO) analysis of DEGs. (**D**) Kyoto Encyclopedia of Genes and Genomes (KEGG) analysis of DEGs. (**E**,**F**) PPI and hub genes analysis of DEGs.

Firstly, we compare the different genes in the TARGET and GTEx datasets by setting |Log_2_FC| > 2, P < 0.05 as the threshold to screen the DEGs. The adjusted P value is based on the FDR Benjamini and Hochberg (BH) correction method. The volcano map of DEGs was made for visualization. Blue and red plots represent down-regulated and up-regulated genes (Fig. 8A). Then we screened NRGs based on DEGs and obtained functional enrichment results through GO analysis. The top five effects of enrichment according to p-value were as follows: regulation of apoptotic signaling pathway, positive regulation of the cellular catabolic process, positive regulation of apoptotic signaling pathway, regulation of cysteine-type endopeptidase activity and positive regulation of catabolic process (Fig. 8B). We found that the maximum enrichment result of the BP is regulation of the apoptotic signaling pathway and that of CC is focal adhesion. The maximum enrichment result of MF is heat shock protein binding (Fig. S3). The KEGG pathway analysis studies the enrichment of signal pathways. The top five KEGG enrichment pathways include salmonella infection, NOD-like receptor signaling pathway, pathogenic Escherichia coli infection, apoptosis and necroptosis. In addition to the above pathway processes, some immune-related pathways, such as Th17 cell differentiation also enriched (Fig. 8D). To supplement and verify the results, NRGs were analyzed at Metascape. Consistent results were shown for GO analysis (Fig. 8C). PPI and Hub genes were predicted and visualized (Fig. 8E,F).

## Discussion

In this study, the NRGs were selected by NMF consistent clustering and LASSO algorithm Cox regression analysis to establish a prediction model by analyzing the osteosarcoma patient datasets from the TARGET and GEO databases GEO database was used for verification. The predictive analysis of clinical indicators was carried out based on the model. Finally, the possible mechanism was discussed by functional enrichment analysis. We have preliminarily concluded that maybe it is a new promising research direction for the early diagnosis and treatment of osteosarcoma disease by NRGs.

Osteosarcomas occur in children and adolescents, are the most common bone and soft tissue malignancies and are difficult to treat^30,31^. Conventional surgery, radiotherapy and chemotherapy still fail to improve survival^8,32^. In recent years, targeted therapies, including targets related to apoptosis, autophagy and ferroptosis, have been gradually recognized, but there are still reports of drug resistance^33–38^. Therefore, the search for new diagnostic and therapeutic targets continues unabated. Eight NRGs were identified by Cox regression analysis. MYCT1, MYC Target 1, is mainly found in the nucleus. Overexpression can cause the promotion of apoptosis, alteration of morphology, enhancement of anchorage-independent growth, tumorigenic conversion, promotion of genomic instability and inhibition of hematopoietic differentiation. It has been reported that MYCT1 is associated with proliferation, transformation and genomic instability of tumors^39^, the full name for BNIP3L is BCL2 Interacting Protein 3 Like. The protein directly targets mitochondria and causes apoptotic changes, including loss of membrane potential and the release of cytochrome C^40^. LRP1 stands for LDL Receptor Related Protein 1. Several cellular processes include intracellular signaling, lipid homeostasis and clearance of apoptotic cells in Alzheimer’s disease^41^. OPTN is short for Optineurin. Optineurin interacts with adenovirus E3-14.7 K protein and may reveal tumor necrosis factor-alpha or Fas-ligand pathways to mediate apoptosis, inflammation, or vasoconstriction^42^. TRIP6 stands for Thyroid Hormone Receptor Interactor 6. It may contribute to adherens junction and promote actin cytoskeleton, cell invasiveness and migration mediates transcription factors NF-Kappa-b and JUN act on inflammatory pathways^43^. ATF4 stands for Activating Factor 4. This gene encodes a transcription factor initially identified as a widely expressed mammalian DNA binding Protein; related to this gene include DNA-binding transcription factor activity and protein heterodimerization activity^44^. TNFRSF1A is TNF Receptor Superfamily Member 1A. It plays a role in cell survival, apoptosis and inflammation^45^. CLTCL1 stands for Clathrin Heavy Chain Like 1. It is a clathrin-heavy chain family member and encodes a major protein of the polyhedral coat of coated pits and vesicles. Related to this gene include binding and structural molecule activity^46^. The first six genes had a positive regulatory effect, while the last two were negative. We explored its possible upstream and downstream mechanisms through PPI and hub genes prediction, which provided a direction for further mechanism exploration.

We found different tumor immune microenvironment outcomes in different groupings in two data sets modeled by NRGs. In the results of NMF clustering in the TARGET dataset and other risk score groups, we found that the tumor microenvironment score in cluster 2 and the low-risk group had a higher stromal score, immune score and ESTIMATE score but lower tumor purity. And this was positively correlated with the prognosis and survival of patients (different risk score groups). Similar prognostic results were found in the GEO data validation set, but no significant statistical differences were found in tumor microenvironment scores. This may be related to the data sample size and other factors. However, NRGs have been reported to influence the tumor microenvironment^47,48^. In addition, tumor microenvironment and immune cells have influenced prognosis in different tumors^49,50^. Additionally, univariate and multivariate Cox regression analyses were performed to investigate the effects of other clinical indicators, such as age, sex, metastasis and NRG risk score, on tumor prognosis. The results were verified by nomogram using TARGET and GEO data. We found that metastasis and risk score were independent risk factors for the poor prognosis of osteosarcoma. This further supports our hypothesis that NRGs and clinical markers can be predictors of osteosarcoma prognosis. Therefore, we can still hypothesize that NRGs may influence tumor prognosis by regulating tumor immune cell microenvironment infiltration. Further experiments are needed to confirm this.

We then compared the differences between osteosarcoma and normal muscle tissue and screened for NRGs for functional enrichment analysis. The enrichment results mainly focus on regulating the apoptotic signaling pathway, positive regulation of the cellular catabolic process and focal adhesion heat shock protein binding. The KEGG pathway analysis includes salmonella infection, nod-like receptor signaling pathway, pathogenic Escherichia coli infection, apoptosis and necroptosis. In addition to the above pathway processes, some immune-related pathways, such as Th17 cell differentiation also enriched. These results also focus on apoptosis and inflammation, consistent with our findings, compared with NRGs functional enrichment in other tumors^20,48,49^. These results demonstrate that osteosarcoma and normal tissue differ in genes associated with cell death and contribute to activating inflammatory, apoptotic and necroptosis signaling pathways.

There is also some limitation in this study. First, due to the restriction of database data, the clinical predictive data of normal control tissues are lacking, so it is impossible to conduct further analysis from clinical indicators such as osteosarcoma stage. Second, this study is mainly based on bioinformatic analysis and lacks experimental data, so its credibility still needs to be verified by further experiments. Third, we have not explored and confirmed the potential mechanism. Thus, the molecular mechanism should be studied in vitro and in vivo. In addition, our study lacks validation of local cohorts and immunotherapy response analysis to validate further and explore immunotarget therapy for necroptosis-related genes. Therefore, we will improve the defects of this part of the work in the future.

## Conclusion

In conclusion, we found a significant role in correlating necroptosis and immunity-promoting osteosarcoma. It may provide a novel insight into detecting molecular mechanisms and targeted therapies for osteosarcoma.

## Supplementary Information

Supplementary Figures.

## Data Availability

The dataset of osteosarcoma was from the TARGET database (Therapeutically Applicable Research to Generate Effective Treatments; https://ocg.cancer.gov/programs/target) and the dataset GSE21257 was from the GEO database (Gene Expression Omnibus; https://www.ncbi.nlm.hih.gov/gds). The dataset of normal muscle tissue was obtained from GTEx (Genotype-Tissue Expression; https://xenabrowser.net/).

## Abbreviations

NRGs: Necroptosis-related genes
DEGs: Different expression genes
TARGET: Therapeutically applicable research to generate effective treatments
GEO: Gene expression omnibus
GTEx: Genotype-tissue expression
NMF: Nonnegative matrix factorization
LASSO: Least absolute shrinkage and selection operator
KM: Kaplan–Meier
ROC: Receiver operating characteristic
TIME: Tumor immunity microenvironment
GO: Gene ontology
KEGG: Kyoto encyclopedia of gene and genomes
BP: Biological process
CC: Cellular components
MF: Molecular function
PPI: Protein–protein interaction
