# Supplementary material for: Construction and validation model of necroptosis-related gene signature associates with immunity for osteosarcoma patients
Source: Sci Rep. 2022 Sep 23;12:15893. doi: 10.1038/s41598-022-20217-4 (PMC9508147; doi:10.1038/s41598-022-20217-4)
Supplement: Supplementary file 1 — Supplementary Figures. [file 41598_2022_20217_MOESM1_ESM.docx]

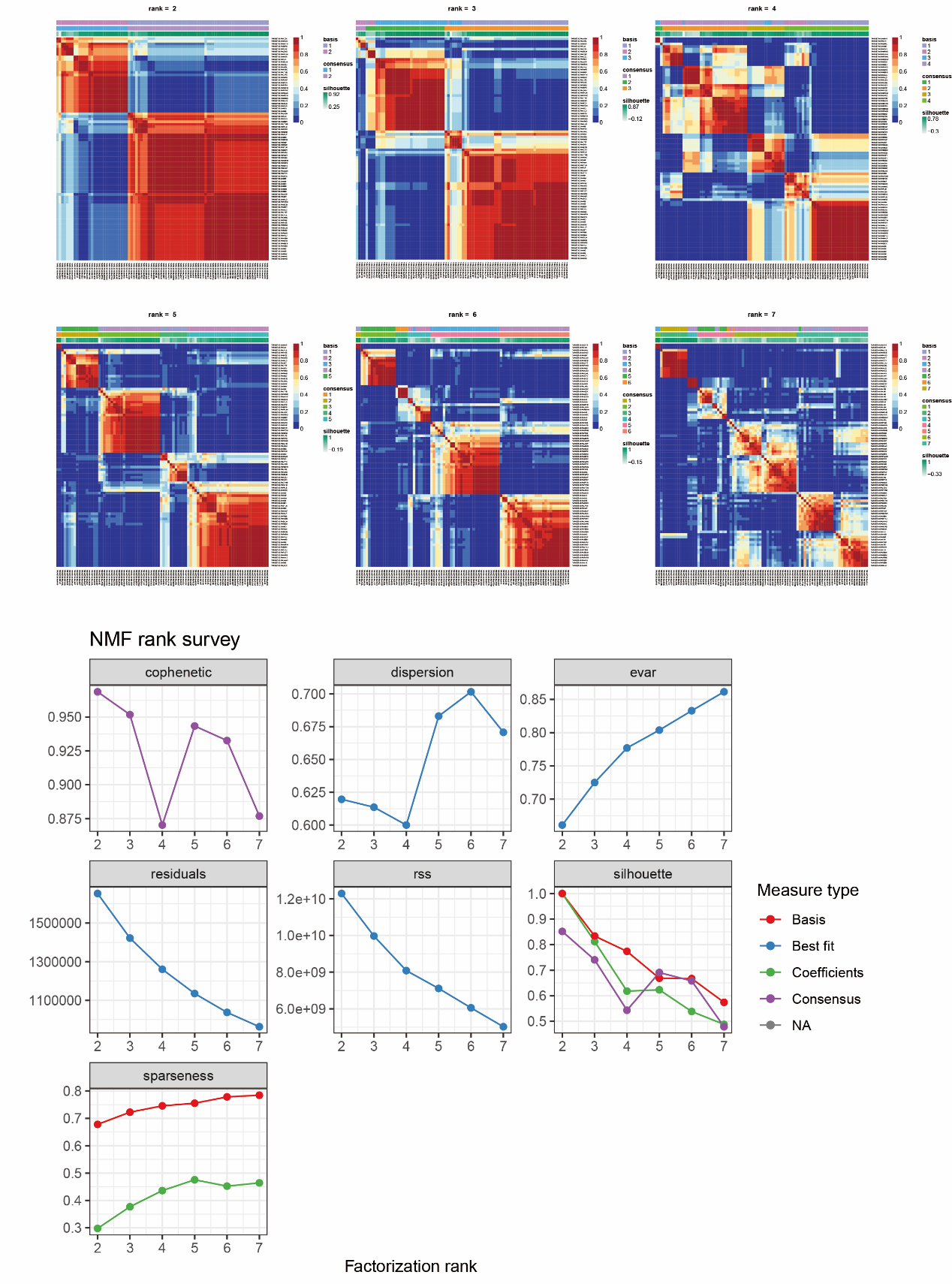


**Figure S1.** NMF rank survey of the clusters.


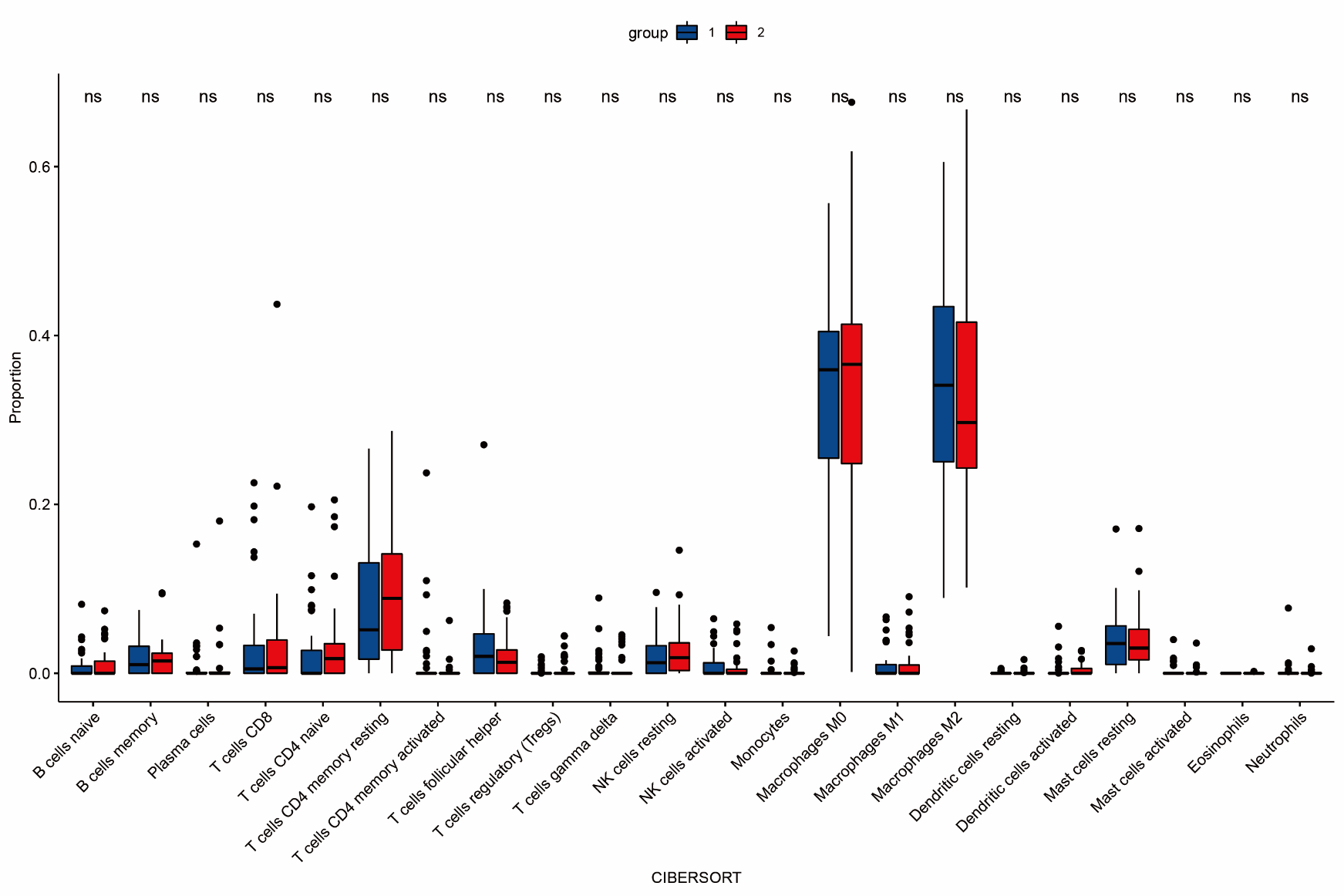


**Figure S2.** Inflammatory cell infiltration analysis by CIBERSORT.


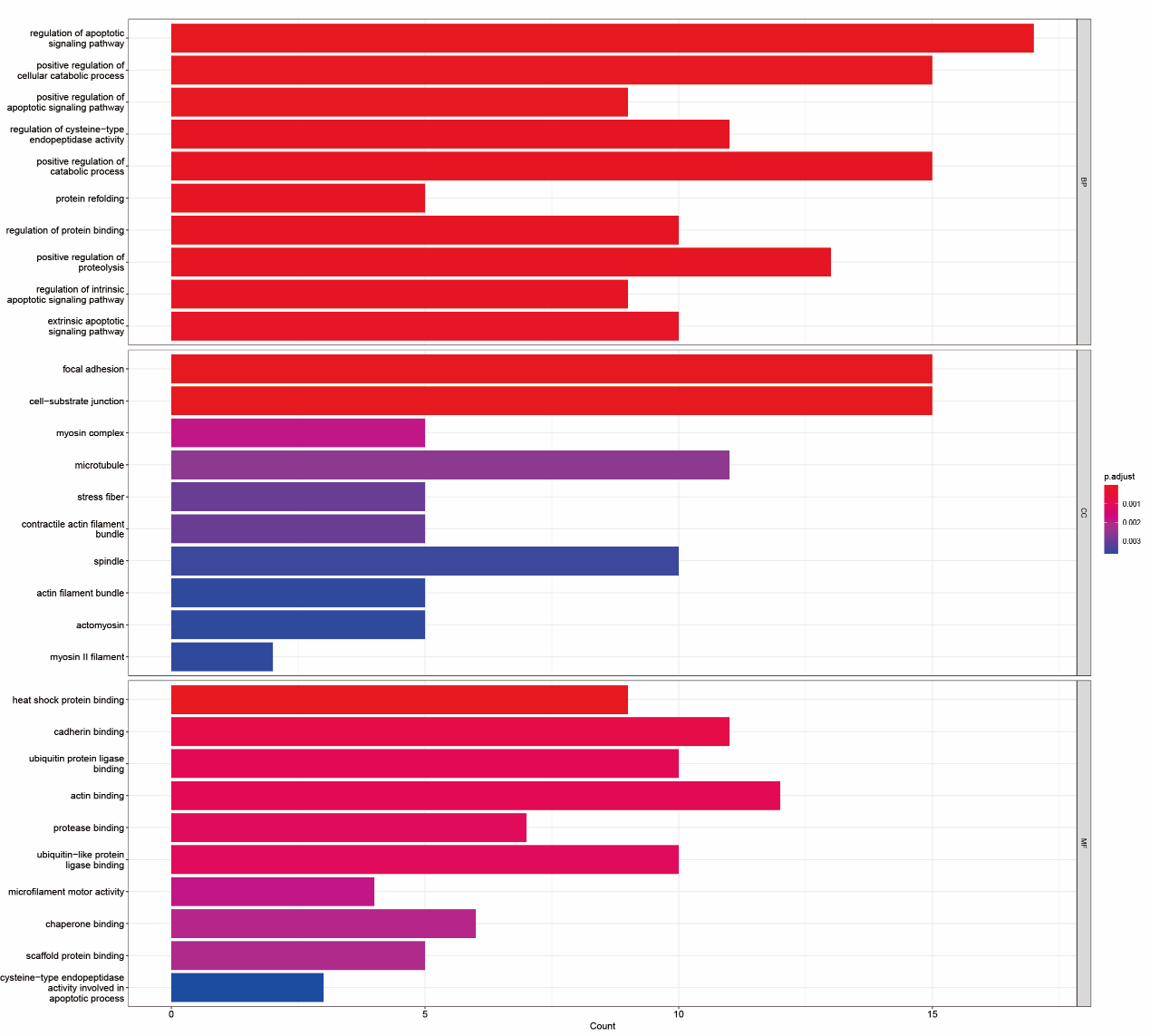


**Figure S3.** GO analysis based on BP, CC, and MF.
